# Individual differences in the neural architecture in semantic processing

**DOI:** 10.1038/s41598-023-49538-8

**Published:** 2024-01-02

**Authors:** Xin Liu, Yiwen Hu, Yaokun Hao, Liu Yang

**Affiliations:** Air Force Medical Center, Air Force Medical University, No. 28, Fucheng Street, Haidian District, Beijing, 100142 China

**Keywords:** Language, Reading

## Abstract

Neural mechanisms underlying semantic processing have been extensively studied by using functional magnetic resonance imaging, nevertheless, the individual differences of it are yet to be unveiled. To further our understanding of functional and anatomical brain organization underlying semantic processing to the level of individual humans, we used out-of-scanner language behavioral data, T1, resting-state, and story comprehension task-evoked functional image data in the Human Connectome Project, to investigate individual variability in the task-evoked semantic processing network, and attempted to predict individuals’ language skills based on task and intrinsic functional connectivity of highly variable regions, by employing a machine-learning framework. Our findings first confirmed that individual variability in both functional and anatomical markers were heterogeneously distributed throughout the semantic processing network, and that the variability increased towards higher levels in the processing hierarchy. Furthermore, intrinsic functional connectivities among these highly variable regions were found to contribute to predict individual reading decoding abilities. The contributing nodes in the overall network were distributed in the left superior, inferior frontal, and temporo-parietal cortices. Our results suggested that the individual differences of neurobiological markers were heterogeneously distributed in the semantic processing network, and that neurobiological markers of highly variable areas are not only linked to individual variability in language skills, but can predict language skills at the individual level.

## Introduction

The human brain is individually unique in neuroanatomy and function, reflected in variability in human cognition and behavior^1–5^. However, these individual differences are often overlooked. This is mainly for reasons of statistical power that require group averaging to improve the signal-to-noise ratio (SNR). This is understandable, but at the same time unfortunate. Characterizing variability in brain function and anatomical structure is important for understanding associations between brain activity and behavior, genetic or personality traits. In recent years, individual differences have drawn much more attention in brain imaging research and an increasing number of studies popped up. Specifically, evidence from multiple independent efforts has demonstrated that there was marked individual variability in neuroanatomy, like anatomical connectivity^6^ and cortical morphological features, but also in functional network architectures under task-free resting-state^5,7,8^. The findings of studies concerning individual differences in the human brain can be categorized into three streams. One stream of resting-state results demonstrated remarkable inter-individual variability in the functional network, and it indeed can serve as a fingerprint for individual identification^8–11^. A minor stream of studies focused on characterizing individual variability in anatomical^4^ and functional markers^1,12,13^ distribution in the whole brain. Mueller et al. (2013) demonstrated that individual differences in functional connectivity were heterogeneous across the cortex, with significantly higher variability in heteromodal association cortex and lower variability in unimodal cortices^1^. This finding is also confirmed by studies investigating individual differences in anatomical markers^4^. Langs et al. (2016) found that the spatial distribution of association networks, including the frontal, parietal, and temporal regions was highly variable, and the unimodal areas, including the motor, sensory, and visual cortices, showed minimal spatial distribution variability. Another major stream of studies concerning individual differences in neural basis focused on linking human cognitive ability to neurofunctional^14–17^ or neuroanatomical markers^18–20^. For example, there is evidence showing vocabulary size was positively correlated with neural efficiency (less activated voxel amounts) in the right hemisphere homologues of language regions in reading narratives^17^, and working memory was positively correlated with neural adaptability in inferior occipital and temporal regions in reading sentences with increasing lexical processing load^16^. In addition, Cui et al. demonstrated that the cortical representation of association networks could predict individual differences in executive function^20^, and the gray matter volume covering the putative, cerebellum, and subcortical systems could predict individual differences in reading comprehensive ability^18^.

As described above, studies of resting-state functional MRI explored intersubject variability in functional connectivity, and demonstrated the individual differences in functional connectivity were heterogeneous across the cortex, with significantly higher variability in heteromodal association cortex and lower variability in unimodal cortices in both health^1,3^ and preterm human brain^2^. However, until now, only a few studies focused on individual differences in the neurobiology of language processing^12,13,15,16,18^. A stream of these studies focused on characterizing individual variability in functional markers distribution in the brain mechanism evoked by language tasks^12,13^. For example, Seghier et al. (2004) examined the individual variability of brain activity location in a word rhyming detection and a word semantic judgment task. They demonstrated that the inferior frontal gyrus, temporal regions and occipito-parietal regions demonstrated a pronounced variability across individuals, while motor regions showed less variability^13^. In addition, Ren et al. (2021) focused their interests on brain activity evoked by a passively listening to vocal and nonvocal stimuli task, they observed lower individual variability in task-evoked activity (during vocal and non-vocal sounds listening) in Heschl’s gyrus, but higher variability in the lateral superior temporal gyrus^12^. These studies seem to support the idea that task-evoked functional variability increases in later stages of the processing hierarchy; that is, more individual variation is observed in the higher-level association cortices than in the lower-level sensorimotor regions. However, these studies focused their interests on lexical or phonological processing level, and on characterizing individual differences in functional markers only. We are still unclear about the individual variability distribution of anatomical and functional markers in the brain mechanism induced by different language processing components. The other stream of studies focused on linking human language ability to neurofunctional or neuroanatomical markers^15,16,18^. However, these studies have focused primarily on the neural regions with the highest overall activity during tasks, instead of operationalizing “regions of variance” as regions of interest. It has been shown that “regions of variance”, which are brain regions with the most functional variability across individuals, were more predictive of individual differences in personality with affective music listening, compared to regions that were highly activated in group-level whole-brain analysis^21^. Therefore, the significance of highly variable brain regions to individual cognition remains to be extensively investigated, and a proper understanding of the neural underpinnings of the language process requires an appreciation of the degree to which language-related activations vary across individuals.

Language is a complex cognitive processing mechanism, which is composed of multiple building blocks. Basic building blocks include the knowledge that has been acquired during development about the sound patterns of the one or more languages a speaker commands, the meaning of its lexical items, their syntactic features, the orthographic patterns, or the signs in the languages of the deaf^22^. In these building blocks, the cognitive act of accessing stored knowledge about the world is the semantic processing of a language^23^. The neural basis of semantic processing has been extensively studied by brain imaging research, and relied on a network of brain areas spanning the frontal, inferior parietal, and temporal cortices^23,24^. These regions form a hierarchically ordered neural ensemble, with inferior parietal and ventral temporal cortices acting as high-level semantic convergence zones. In contrast, the temporal pole and superior temporal cortex form low-level modality-specific sensory and motor systems^23,25^. There are studies demonstrating that the neural underpinnings of language are characterized by individual differences^13,15–17,26,27^. However, individual differences in the semantic processing brain mechanism still have been a largely under-explored domain.

Overall, individual differences distribution in brain anatomical and functional markers which were induced by semantic tasks are still poorly understood. Meanwhile, the significance of highly variable brain regions to individual language experiences remains to be extensively investigated. To address these issues, in our current study, we aimed to investigate how the variability of task-evoked brain activity and grey matter volume is distributed in a story comprehension network. This we did by leveraging T1 and functional image data of a story comprehension task^24^. This task was part of the Human Connectome Project (HCP)^28^. In this task, participants were instructed to either listen to a brief story and then answer subsequent comprehension questions about the story topic (story condition), or listen to arithmetic operation problems and indicate the correct answer (math condition). The story condition was implemented to engage rapid integration of conceptual information. Hence it induced activation in especially the inferior frontal and temporo-parietal cortex. Conversely, the math condition used verbal, sentence-like stimuli that were matched to the stories in terms of low-level auditory or phonological features. Therefore, the story minus math contrast was expected to elicit a network of brain clusters that were thought to be responsible for semantic processing^24^. We expected that the high-level semantic processing areas (inferior parietotemporal cortex and prefrontal cortex) would show greater individual differences than modality-specific auditory processing and sensorimotor regions, both in task-evoked functional activity and grey matter volume. In addition, we further investigated whether behavioral language skills can be explained and predicted by functional networks which were composed of regions with substantial individual differences. We hypothesized that individual variability in language skills would be predicted by brain regions with high individual variability.

## Methods

### Participants

We used language behavioral test scores and neuroimaging data from the 1200 Subjects Data Release of the HCP dataset^28^ (see https://www.humanconnectome.org/study/hcp-young-adult/document/1200-subjects-data-release). We focused our analyses on the 100 Unrelated Subject Release (see https://db.humanconnectome.org/), which is a subset of the 1200 Subjects Data Release. All participants are non-sibling, in order to remove the influence of family-genetic factors on individual differences in brain function and structure. Of these 100 subjects, three with head movement parameters greater than 2.5 mm of displacement or 2.5 degrees of rotation in any direction were excluded from further analyses. Thus, our final sample contained 97 participants (age = 22–35; 44 males; see in Table 1) who completed the 3T MRI protocols and had available data of the language task fMRI, resting-state fMRI, and T1 registration, as well as behavioral measures of language tests.

**Table 1:** The demographic, cognitive, and behavioral characteristics of participants.

	Characteristic	Mean (range)
Demographics	Age	28.50 (22–35)
	Gender (male, %)	45.36
Language tests	Vocabulary comprehension (PVT)	115.22 (92.04–134.24)
	Reading decoding (ORRT)	115.82 (86.20–135.81)
In-scanner behavior	Accuracy (%)	95.36 (62.50–100.00)
	Reaction time (ms)	3342 (2711–4477)

*PVT:* Picture Vocabulary Test; *ORRT:* Oral Reading Recognition Test. Accuracy and Reaction time refer to behavioral performance in story blocks.

### Task

We focused on the language task fMRI session in HCP dataset, which was developed by Binder et al. (2011)^24^. The task consisted of two runs, each interleaving 4 blocks of a story condition and 4 blocks of a math condition. As described in detail in Binder et al. (2011)^24^, the story blocks presented participants with brief auditory fables (5–9 sentences), followed by a two-alternative forced-choice question about the topic of the fable. During the math blocks, participants were aurally presented with a series of arithmetic operations trials, which were designed to match the length of the story task blocks. They were also completed with two-alternative questions asking for the correct answer to the math trials. Average response accuracy and reaction times of the story blocks are shown in Table 1.

### Language tests

The HCP dataset includes two behavioral tests assessing language skills, both taken from the NIH toolbox^29^, focusing on two aspects of language. The first task probes receptive word knowledge and vocabulary comprehension skills, which was measured by a picture vocabulary test (PVT), in which participants were orally given a word and were instructed to select the best matching picture from four given pictures on a screen. The second task tapped into reading decoding skills, by using an Oral Reading Recognition Test (ORRT). Participants were asked to pronounce single printed letters or words, including words that occur infrequently and have irregular orthography. It is reflective of the level and quality of prior educational experiences^30^. Ninety-seven participants’ overall performance of these two tasks were shown in Table 1.

### Image acquisition

In the HCP dataset, whole brain high-resolution (2.0 mm isotropic voxels) fMRI images were acquired using a customized Siemens Skyra 3-T scanner with a 32 channel head coil. A gradient echo EPI sequence was used with the following imaging parameters: TR = 720 ms, TE = 33.1 ms, flip angle = 52°, FOV = 208 × 180 mm, slice thickness = 2.0 mm, 72 slices, with a multi-band acceleration factor of 8.

Structural scans included T1w and T2w scans. Parameters of T1w structural scans were: TR = 2400 ms, TE = 2.14 ms, flip angle = 8°, FOV = 224 × 224 mm, voxel size = 0.7 mm. T2w structural scans were acquired using TR = 3200 ms, TE = 565 ms, FOV = 224 × 224 mm, voxel size = 0.7 mm, with a variable flip angle. See WU-Minn HCP manual and HCP scan protocols present an overview of the MRI acquisition details (https://www.humanconnectome.org/study/hcp-young-adult/document/1200-subjects-data-release).

### MRI data pre-processing

Preprocessing of functional and structural scans was performed using the HCP minimal preprocessing pipeline including artifact removal, motion correction and registration to standard space^31^. Three additional approaches were used for further network construction of the fMRI data. One involved nuisance regression, this step was carried out for the task fMRI only, 24-parameter motion regressors (6 head motion parameters, 6 head motion parameters one time point before, and the 12 corresponding squared items), average time-series from the cerebrospinal fluid (CSF) and the white matter were regressed out. The second is global signal regression, involving the removal of the global signal from the time series of each voxel using linear regression, which was applied for both fMRI modalities. The last is bandpass filtering, we bandpass filter the time-series with the minimum frequency of 0.009 Hz, maximum frequency of 0.08 Hz for resting state and 0.25 Hz for task state fMRI. The length of the language task fMRI scans is 316 TRs, which does not correspond to the 1200 TRs in the resting-state data. To rule out the effects of scan length, we restricted the resting state analysis to the first 316 volumes in each run.

### fMRI data analysis

#### Collective whole-brain local activity analysis

In the first-level model, the preprocessed functional volumes were submitted to a general linear modal (GLM) with two conditions: story and mathematical calculation respectively. The BOLD response for each condition was modeled with the canonical hemodynamic response function, along with its temporal derivative. Six parameter estimates of head motion were entered as confounding regressors to correct for potential movement artifacts. A one-sample t-test was applied in the second-level analysis to generate statistic inferences of semantic processing, by contrasting images of the story condition minus the math condition.

#### Individual activity variance analysis

Based on the collective semantic processing network, we investigated how individual differences in brain activity were distributed throughout this network by carrying out an individual activity variance analysis. We calculated a variance map across individuals for the story minus math contrast. The variance map was based on the ratio of between-individuals variance to within-individuals variance, which was calculated for each voxel in the story minus math contrast, using the following formula^32^:

1 $$S_{B}^{2} = { }\frac{{\frac{1}{NSubj - 1 }\mathop \sum \nolimits_{i = 1}^{NSubj} \left( {con*.img_{i} - \overline{con*.img} } \right)^{2} }}{2} \times NScan.$$

2 $$S_{W}^{2} = \frac{{\frac{1}{NSubj}\mathop \sum \nolimits_{i = 1}^{NSubj} ResMS.img_{i} }}{NScan - 1}.$$

3 $$F = \frac{{S_{B}^{2} }}{{S_{W}^{2} }}.$$

where, ${S}_{B}^{2}$ is the between-individuals variance, ${S}_{W}^{2}$ is the within-individuals variance, *F* is the ratio of between- to within-individuals variance, *NSubj* is the number of subjects, *NScan* is the number of scans, ${con*.img}_{i}$ is the voxel value from the ith subject’s contrast image, $\stackrel{-}{{con*.img}_{i}}$ is the mean voxel value across subjects from the contrast image, ${ResMS.img}_{i}$ is the image of the mean squared residual (mean across time) for the given participant.

The resultant F-map was used to estimate the individual variance in activity under story minus math contrast. The value of each voxel in the F-map indicated the degree to which the between-individuals variance was larger than the within-individuals variance. The F-map was then subjected to a significance thresholding method using the *findchangepts* algorithm^21^. Concretely, voxels in the F-map were sorted in a vector according to their F-value, and then the “change-point” in the vector was determined as an arbitrary significant threshold. The sorted F-values for the story minus math contrast and the respective change point (1.85 × 10^6^) is shown in Supplementary Fig. 1. Thus, the F-map was thresholded at the voxel level using this change-point as the threshold. In this way, only voxels were kept whose F-value exceeded the statistical threshold of 1.85 × 10^6^. Voxels whose F-values were below this threshold were given a value of 0. This thresholded F-map was then applied to the second-level binary T-map to generate an activity variance map for the semantic processing network.

### sMRI data processing

#### Individual grey matter volume variance analysis

In order to investigate how individual differences in grey matter volume were distributed throughout the semantic processing network, we implemented an individual grey matter volume variance analysis. The individual grey matter volume variance map was estimated by calculating the standard deviation (SD) of voxels in GM volume images across individuals. Similarly to the individual activity variance analysis, this grey matter volume variance map was thresholded using the *findchangepts* algorithm^21^. Specifically, all the values in the individual grey matter volume variance map were sorted, and the abruptly changed point in the sorted list was defined as the threshold. The resultant threshold is 0.07. The sorted SD values of grey matter volume and the relevant threshold can be found in Figure S1. Voxels for which the SD value exceeded 0.07 were regarded as significant. Voxels with a value below this threshold were set to 0 and excluded from further analysis. This thresholded grey matter volume variance map was projected onto the second-level binary T-map mask, and a grey matter volume variance map for the semantic processing network was obtained.

### Brain-behavior correlation analysis

In order to establish relationships between brain measurements and behavior performance, we extracted local functional activity or grey matter volume of regions of variance (ROVs) as two neurobiological markers and carried out correlation analyses to relate them with language skill measures respectively. ROVs were selected based on the clusters significantly varied in functional activity or grey matter volume. Specifically, functional ROVs (fROVs) were defined as 10 mm spheres around the peak coordinates in MNI space of clusters showing significant activation variance. Six fROVs were identified, which were located in right superior medial frontal gyrus (RSFGmed, BA 9, coordinates [x y z] = [9 51 39]), left angular gyrus (LANG, BA 39, [− 42 − 54 24]), left inferior frontal gyrus, pars opercularis (LIFGoperc, BA 44, [− 54 18 18]), right superior temporal gyrus (RSTG, BA 40, [54 − 24 15]), left precuneus (LPCUN, BA 31, [− 3 − 60 36]), and left superior temporal gyrus (LSTG, BA 41, [− 39 − 36 12]). In addition, seven regions were defined as structural ROIs (sROVs). Again, they were defined as 10 mm spheres around the peak coordinates in MNI space of clusters showing significant grey matter volume variance, located in the left dorsolateral superior frontal gyrus (LSFGdor, BA 6, [− 20 16 45]), right inferior frontal gyrus, pars triangularis (RIFGtriang, BA 45, [39 18 27]), left inferior frontal gyrus, pars triangularis (LIFGtriang, BA 44, [− 36 15 27]), left angular gyrus (LANG, BA 39, [− 39 − 57 30]), right angular gyrus (RANG, BA 39, [45 − 48 27]), right precentral gyrus (RPreCG, [18 − 18 66]), and right caudate nucleus (RCAU, [15 6 21]). Weighted parameter estimates (beta weights) of the story minus math contrasts of the six fROVs were extracted as functional markers, and the grey matter volume values of the seven sROVs were extracted as anatomical markers. The measures of the participants’ language skills were first checked to be normally distributed, and outliers were replaced by the mean values. After this, the functional markers of the six fROVs were correlated to these measures using Pearson correlation. Likewise, the anatomical markers of the seven sROVs were also correlated to the language skill measures after controlling for brain size variance. All resulting *p* values were corrected for multiple comparisons using Bonferroni correction.

### Brain-behavior prediction analysis

#### Network construction

**Figure 1:**
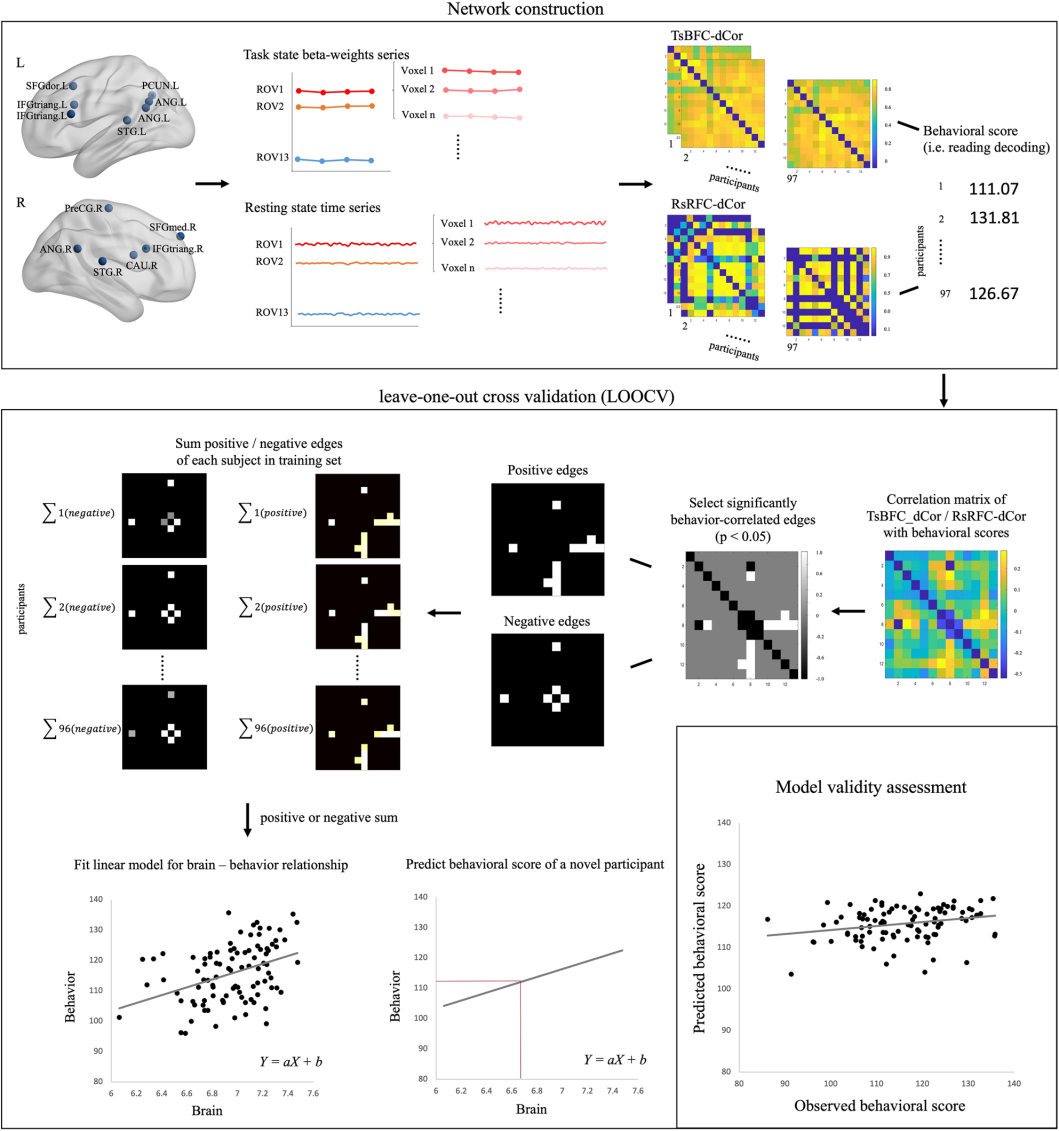
The schematic work flow of connectome-based predictive modeling. Network construction. All voxels’ beta-weights series (in task state) and raw time series (in resting state) in each of the thirteen ROVs were extracted and normalized. Then, for each participant, a task-evoked beta-weights network and a resting-state raw time series network were calculated by Euclidean distance correlation, which were named as TsBFC-dCor and RsRFC-dCor, both were 13 × 13 symmetric FC (functional connectivity) matrices. In addition, each participants’ behavioral score were prepared for predictive model construction and validation. SFGdor: dorsolateral superior frontal gyrus; IFGtriang: inferior frontal gyrus, triangle part; IFGoperc: inferior frontal gyrus, opercular part; PCUN: precuneus; ANG: angular gyrus; STG: superior temporal gyrus; PreCG: precentral gyrus; CAU: caudate nucleus; SFGmed: superior median frontal gyrus. L: left; R: right. Leave-one-out cross validation. We used a leave-one-out cross validation (LOOCV) to train and test behavior prediction model. First, behaviorally relevant edges were identified by performing Pearson correlations between each FC strength in FC matrices (TsBFC-dCor or RsRFC-dCor) and behavioral scores (i.e. reading decoding) in the training set of 96 participants. Next, only the most significant edges were selected as predictive edges (*p* < 0.05), resulting a group-level 13 × 13 matrix, in which each edge represented the degree of correlation between these FC strength and the behavioral scores. Later, group-level matrices were divided into a positive network (i.e., positive correlations between behavioral scores and FC matrices) and a negative network (i.e., negative correlations between behavioral scores and FC matrices). The example given here indicated the existence of both positive and negative networks, but there might be only a positive or a negative network survived in the actual correlation between FC networks and behavioral scores (e.g., in the correlation matrix between resting-state FC networks and reading decoding scores, there is no negative network observed). Next, two separate binary masks (with 1 representing a significant correlation between this FC strength and behavior and 0 representing a non-significant correlation) were generated and multiplied to the individual FC matrix to generate two series of behaviorally positive and negative related matrices. We then summed the edges of the positive and the negative matrices separately for each participant respectively. The summed edges’ values and behavioral scores across the training set were used to fit a linear model, resulting a positive and negative model respectively. These models were then applied to predict a novel participant’s behavioral score according to his summed FC network edges. Model validity assessment. The model validity was evaluated by Pearson correlation coefficient between predicted and observed behavioral scores for the positive and negative models respectively. A 1000 time permutation test was applied to test the significance of the prediction performance.

Task-evoked and resting-state networks were constructed based on the thirteen aforementioned ROVs as nodes. They were identified to be the regions with the highest inter-individual variability, either in functional activity or in grey matter volume. Unlike traditional resting-state network construction using raw time series as inputs^33–35^, we used the beta-weights of each story trial to construct the task-evoked functional connectivity (FC) matrix. This beta-weights FC is able to measure the absolute connectivity under a specific task condition^36^. In addition, we applied multivariate distance correlation in order to estimate the multivariate correlation between two ROVs and thereby construct the FC matrix. The difference between distance correlation and the traditional univariate Pearson correlation is that the former uses all voxel-wise time series within a node, rather than averaging them^37^. Prior to distance correlation calculation, each voxel’s beta-weights series were extracted and normalized (Z-scored) in each ROV. Then, the Euclidean distance between each pair of time points was calculated for each ROV separately. Afterwards, we applied U-centering to the Euclidean distance matrices, and these centered distance matrices were used to compute the distance covariance and distance variance afterwards. The resultant distance covariance and variance were finally used to calculate the distance correlation for every pairs of the thirteen ROVs, thus resulting in a task-evoked beta-weights network calculated by distance correlation (TsBFC-dCor) for each individual, which is a 13 × 13 symmetric connectivity matrix. The formula and algorithm of the Euclidean distance method can be found in the previous works by Yoo et al. (2019)^37^ and Geerligs and Henson (2016)^38^ in detail. As for the resting-state network construction, we used raw time series as inputs to calculate the FC matrix. Similarly to the task-evoked network, a resting state network (RsRFC-dCor) was generated for each individual by Euclidean distance correlation as well. The schematic construction pipeline can be found in Fig. 1 (Network construction) in more detail.

#### Brain-behavior prediction

We used Connectome-based predictive modeling (CPM) to predict individuals’ behavioral language skills based on the task-evoked and resting-state functional networks, which were obtained in the aforementioned analyses. Prediction models were trained and tested using a leave-one-out cross validation (LOOCV) method. Specially, in each iteration, 96 subjects’ functional networks were used as a training set to construct a predictive model, and the behavioral score of the one remaining subject was predicted by this model. The observed and predicted scores were correlated to estimate the prediction validity. Next, we investigated the feature distribution driving CPM prediction for successfully predicted behavioral language tests.

In the training procedure, we first conducted a Pearson correlation analysis to examine the relevance of each edge in the FC networks to each behavioral score. The resultant r values were then thresholded at *p* ≤ 0.05. Only the edges that passed the threshold were used to build the predictive model. Next, the significant edges were separated into a positive network (i.e., positive correlation between edge strength and behavioral scores) and negative network (i.e., negative correlation between edge strength and behavioral scores). The resultant group-level positive and negative networks were further transformed into two binary masks (with 1 representing a significant correlation between edge strength and behavior and 0 representing a non-significant correlation). Subsequently, these two masks were multiplied to the individual FC networks to generate two behavioral related networks (a positive network and a negative network) for each participants, and the edges of the positive and the negative networks were summed up separately. Finally, we built a linear model by fitting it with the sum of positive or negative edges and behavioral scores in the training set, resulting in a positive model and a negative model respectively. During the testing procedure, the positive model and the negative model were applied to predict a novel participant’s behavioral score. The training and testing procedures were repeated 97 times so each participants’ behavioral score was predicted twice by the positive and negative models which were constructed from the remaining 96 participants’ FC networks. The model’s validity was evaluated by Pearson correlation coefficient between predicted and observed behavioral scores for the positive and negative models respectively. The schematic work flow of the prediction and testing procedure can be found in Fig. 1 (Leave-one-out cross validation) in detail. A 1000 time permutation test was additionally applied to test the significance of the prediction performance.

## Results

### Collective semantic processing network

**Figure 2:**
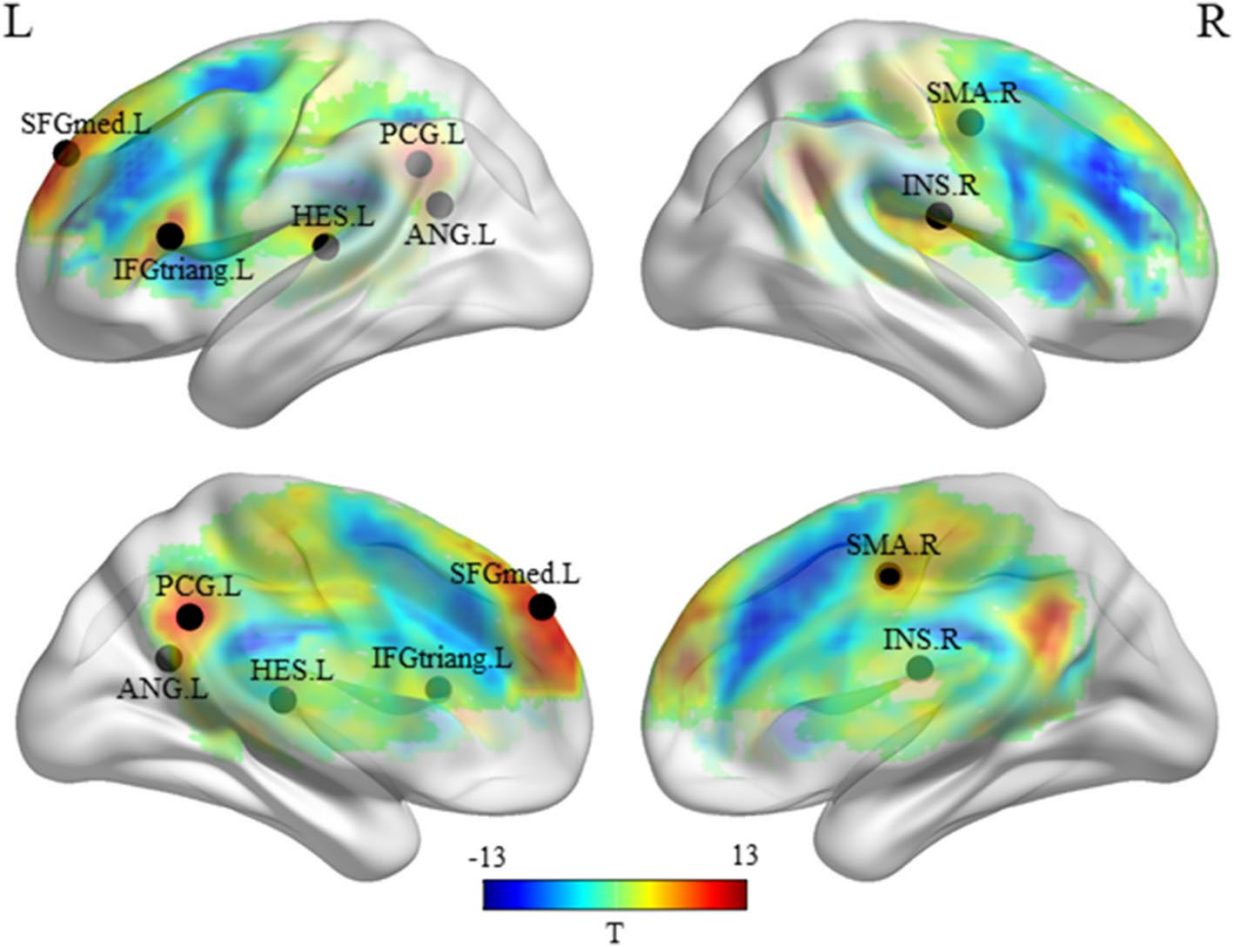
The collective semantic processing network. Univariate T-map contrasted activity during task (story) and baseline (math) images. The color scale linearly reflects the T-value of the contrast between tasks and baselines in voxels above the threshold (FWE-correction, *p* ≤ 0.05, voxel size ≥ 10). Peak-activation of the T-maps for seven clusters are distributed in left SFGmed, PCG, ANG, IFGtriang, HES, and right INS and SMA. SFGmed: medial superior frontal gyrus; PCG: posterior cingulate gyrus; ANG: angular gyrus; IFGtriang: inferior frontal gyrus, pars triangularis; HES: heschl gyrus; INS: insula; SMA: supplementary motor area. BrainNet Viewer (version 1.7) was used to generate image (https://www.nitrc.org/projects/bnv/).

The whole-brain collective analysis revealed seven clusters, which distributed bilaterally in fronto-temporo-parietal regions by comparing activation during the story condition with the math condition. Peak-activation of five clusters were located in left SFGmed, posterior cingulate gyrus (PCG), ANG, IFGtriang and HES, and two clusters were located in right hemisphere, including insula (INS) and supplementary motor area (SMA) (FWE-corrected *p* ≤ 0.05, voxel size ≥ 10). See Table 2 for details and Fig. 2 for lateral and medial views of the statistical T-maps of these results.

**Table 2:** Anatomical locations and stereotaxic coordinates of significant clusters revealed by Story–Math contrast.

Cluster	Size	Anatomical location (AAL)	Anatomical location (BA)	L/R	T	MNI coordinates		
						x	y	z
Prefrontal cortex	474	**SFGmed**	**BA9**	**L**	**12.368**	**− 6**	**51**	**36**
		SFGmed	BA9	L	11.724	**− **6	54	24
		SFGmed	BA9	L	11.218	**− **15	51	33
Post cingulum	218	**PCG**	**BA23**	**L**	**11.666**	**− 9**	**− 51**	**33**
		PCG	BA23	L	11.365	0	**− **48	30
		PCUN	BA31	R	9.742	6	**− **54	33
Posterior inferior parietal lobe	74	**ANG**	**BA39**	**L**	**9.765**	**− 39**	**− 57**	**21**
		SMG	BA40	R	6.984	39	**− **27	18
Insula	102	**INS**		**R**	**9.085**	**39**	**− 15**	**18**
		INS		R	8.696	45	**− **15	12
Inferior frontal gyrus	66	**IFGtriang**	**BA45**	**L**	**8.965**	**− 54**	**21**	**12**
		IFGtriang	BA45	L	8.483	**− **54	27	6
Primary auditory cortex	54	**HES**	**PrimAuditory(41)**	**L**	**8.151**	**− 36**	**− 24**	**9**
		INS		L	8.030	**− **36	**− **21	18
		STG	PrimAuditory(41)	L	6.896	**− **39	**− **36	12
Medial motor cortex	102	**SMA**	**BA6**	**R**	**7.112**	**12**	**− 6**	**45**
		MCG	BA24	L	6.924	**− **3	**− **6	42
		MCG	BA24	L	6.877	0	**− **18	48

Seven clusters were identified to be significant in Story-Math contrast (FWE-corrected *p* ≤ 0.05, voxel size ≥ 10). Peak-values of the T-maps for each cluster were marked in bold.

*Size* number of voxels in the cluster; *AAL:* Automatic Anatomic Labeling atlas; *BA:* Brodmann area; *L/R:* left/right hemisphere; *T:* T-values; *SFGmed:* medial superior frontal gyrus; *PCG:* posterior cigulate gyrus; *PCUN:* precuneus; *ANG:* angular gyrus; *SMG:* supramarginal gyrus; *INS:* insula; *IFGtriang:* inferior frontal gyrus, triang part; *HES:* heschl gyrus; *STG:* superior temporal gyrus; *SMA:* supplementary motor area; *MCG:* median cingulate gyrus.

### Individual differences in functional activity of the semantic processing network

**Figure 3:**
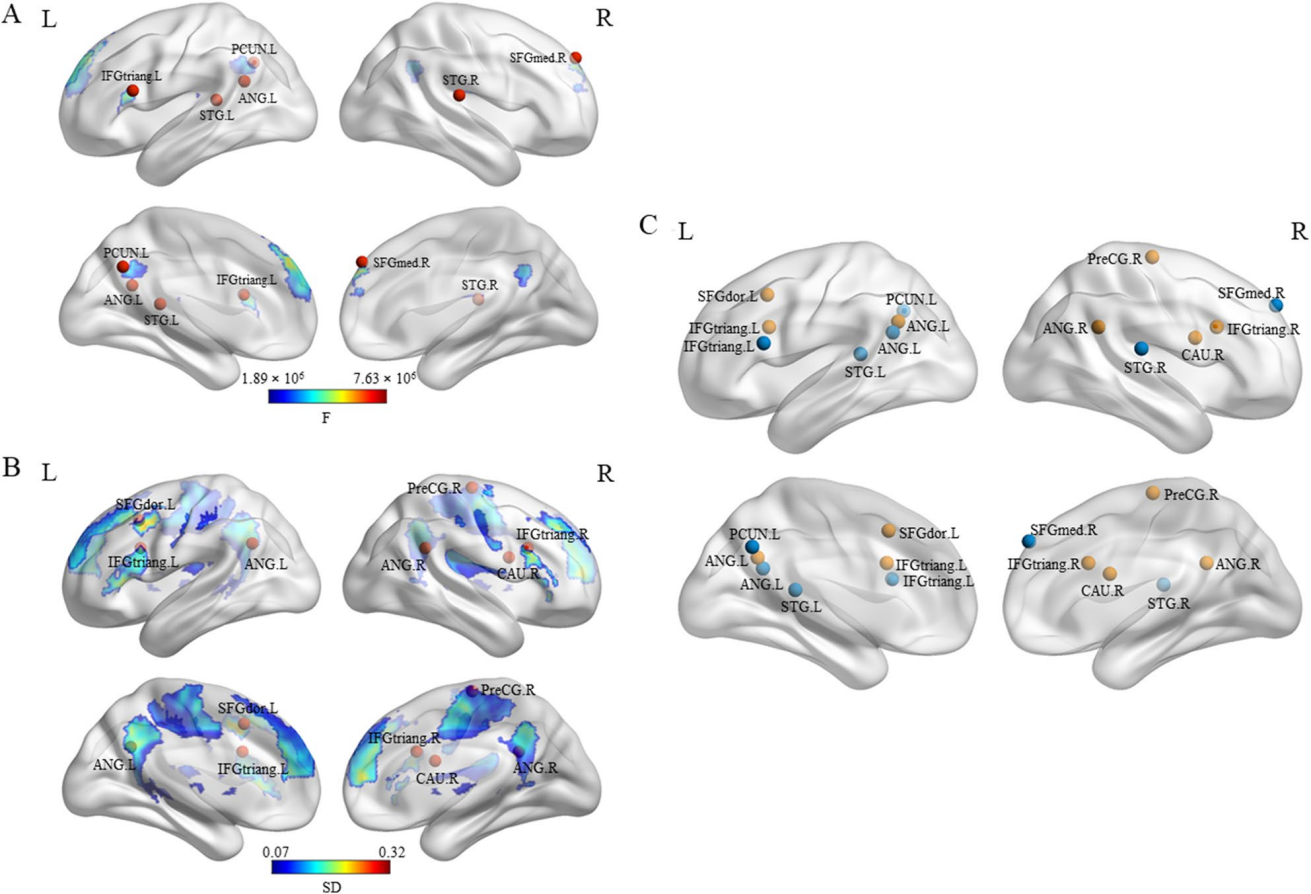
Individual difference distribution of functional activity and grey matter volume in the semantic processing network. (**A**) Activity variance map (F-map) estimating individual differences in functional activity based on task minus baseline contrast. The color scale linearly reflects the F-value estimating the ratio of between-individuals variance to within-individuals variance for each voxel above the threshold (F-value > 1.85 × 10^6^), peaks of six significant clusters in the F map are distributed in right SFGmed, left ANG, IFGtriang, bilateral STG, and left PCUN, which were marked as red nodes. (**B**) Grey matter volume variance map (SD-map) estimating individual differences in grey matter volume. The color scale linearly reflects the standard deviation (SD) of grey matter volume images across individuals (SD-value > 0.07), peaks of seven significant clusters in the SD-map are located in left SFGdor, bilateral IFGtriang and ANG, right PreCG and CAU, which were marked as red nodes. (**C**) Thirteen regions of variance (ROVs). Functional ROVs (fROVs) are in blue, structural ROVs (sROVs) are in orange. SFGmed: medial superior frontal gyrus; SFGdor: dorsalateral superior frontal gyrus; IFGtriang: inferior frontal gyrus, pars triangularis; ANG: angular gyrus; STG: superior temporal gyrus; PCUN: precuneus; PreCG: precentral gyrus; CAU: caudate nucleus. BrainNet Viewer (version 1.7) was used to generate image (https://www.nitrc.org/projects/bnv/).

The individual activity variance map (F-map) can be seen in Fig. 3A (see Table 3 for details). Generally, individual variability in functional activity was heterogeneous throughout the semantic processing network, with significantly higher variability in frontoparietal cortex and lower variability in insula and motor cortices. More specifically, six clusters in the semantic processing network were identified that significantly varied across participants (F-value > 1.85 × 10^6^, FWE-corrected *p* ≤ 0.05, voxel size ≥ 10). The right SFGmed and the left ANG showed the highest individual variance, followed by the left IFGtriang and the right STG. The left cingulate gyrus (precuneus (PCUN) and PCG) and Heschl’s gyrus showed less individual variation, while the right INS and SMA did not show significant variance across individuals, although they were “activated” in the task.

**Table 3:** Anatomical locations and stereotaxic coordinates of clusters of the semantic processing network that showed significant individual variation in functional activity.

Cluster	Size	Peak in AAL	Peak in BA	F (10^6^)	MNI coordinates			Cluster structure (AAL)	Cluster structure (BA)
					x	y	z		
Prefrontal cortex	308	SFGmed.R	BA9.R	7.62	9	51	39	SFGmed.L	BA9.R
								SFGdor.L	BA10.L
								SFGmed.R	BA8.L
Posterior inferior parietal lobe	10	ANG.L	BA39.L	7.32	− 42	− 54	24	ANG.L	BA39.L
								MTG.L	
Inferior frontal gyrus	53	IFGtriang.L	BA45.L	5.93	− 54	18	18	IFGtriang.L	BA45.L
								IFGorb.L	BA44.L
Posterior inferior parietal lobe	35	STG.R	BA40.R	5.30	54	− 24	15	ROL.R	BA13.R
								HES.R	BA41.R
								STG.R	BA40.R
Cingulum	139	PCUN.L	BA31.L	4.72	− 3	− 60	36	PCUN.L	BA31.L
								PCUN.R	BA7.L
								PCG.L	BA23.R
								MCG.L	
								PCG.R	
Primary auditory cortex	21	STG.L	PrimAuditory(41).L	3.01	− 39	− 36	12	ROL.L	BA13.L
								HES.L	BA41.L
								STG.L	

Six clusters were identified that showed significant individual variation (F-value > 1.85 × 10^6^, FWE-corrected *p* ≤ 0.05, voxel size ≥ 10).

*Size* number of voxels in the cluster; *AAL:* Automatic Anatomic Labeling atlas; *BA:* Brodmann area; *L/R:* left/right hemisphere; *F:* F values indicating variance intensity; *SFGmed:* medial superior frontal gyrus; *SFGdor:* dorsalateral superior frontal gyrus; *ANG:* angular gyrus; *MTG:* middle temporal gyrus; *IFGtriang:* inferior frontal gyrus, pars triangularis; *IFGorb:* inferior frontal gyrus, pars orbitalis; *STG:* superior temporal gyrus; *ROL:* rolandic operculum; *HES:* heschl gyrus; *PCUN:* precuneus; *PCG:* posterior cigulate gyrus; *MCG:* median cingulate gyrus.

### Individual differences in grey matter volume of the semantic processing network

Individual differences of grey matter volume in the semantic processing network are shown in Fig. 3B and Table 4. Similar to what was found for the variability in functional activity, individual variability in grey matter volume was heterogeneous as well. The left dorsolateral superior frontal gyrus (SFGdor), bilateral IFG and ANG showed relatively large individual differences, followed by the right sensorimotor areas (pre- and postcentral gyrus), bilateral cingulate cortices (middle cingulate gyrus and precuneus), and the right caudate nucleus (CAU) (SD-value > 0.07, FWE-corrected *p* ≤ 0.05, voxel size ≥ 10). In contrast, left Heschl’s Gyrus and right INS failed to show significant individual variance in grey matter volume.

**Table 4:** Anatomical locations and stereotaxic coordinates of clusters of the semantic processing network that showed significant individual variation in grey matter volume.

Cluster	Size	Peak in AAL	Peak in BA	SD	MNI coordinates			Cluster structure (AAL)	Cluster structure (BA)
					x	y	z		
Prefrontal cortex	1331	SFGdor.L	BA6.L	0.317	− 20	16	45	SFGmed.L/R	BA9.L/R
								SFGdor.L/R	BA10.L/R
								MFG.L	BA8.L
Inferior frontal gyrus	147	IFGtriang.R	BA45.R	0.283	39	18	27	IFGtriang.R	BA45.R
								IFGorb.R	BA47.R
								IFGoperc.R	
Inferior frontal gyrus	255	IFGtriang.L	BA45.L	0.271	− 36	15	27	IFGtriang.L	BA45.L
								IFGoperc.L	BA44.L
								IFGorb.L	BA47.L
Posterior inferior parietal lobe	46	ANG.L	BA39.L	0.257	− 39	− 57	30	ANG.L	BA39.L
								MTG.L	
Posterior inferior parietal lobe	32	ANG.R	BA39.R	0.242	45	− 48	27	ANG.R	BA39.R
								MTG.R	
Sensorimotor	3339	PreCentral.R		0.240	18	− 18	66	MCG.L/R	BA6.R
								PoCG.L/R	BA31.L/R
								PCUN.L/R	BA3.L/R
								PreCG.R	BA4.L/R
								SMA.R	BA24.L/R
Caudate	11	CAU.R		0.113	15	6	21	CAU.R	

Seven clusters were identified that showed significant individual variation (SD-value > 0.07, FWE-corrected *p* ≤ 0.05, voxel size ≥ 10).

*Size* number of voxels in the cluster; *AAL:* Automatic Anatomic Labeling atlas; *BA:* Brodmann area; *L/R:* left/right hemisphere; *SD:* standard deviation values indicating variance intensity; *SFGdor:* dorsalateral superior frontal gyrus; *SFGmed:* medial superior frontal gyrus; *MFG:* middle frontal gyrus; *IFGtriang:* inferior frontal gyrus, pars triangularis; *IFGorb:* inferior frontal gyrus, pars orbitalis; *IFGoperc*: *IFGorb* inferior frontal gyrus, pars oprecularis; *ANG:* angular gyrus; *MTG:* middle temporal gyrus; *MCG:* median cingulate gyrus; *PoCG:* postcentral gyrus; *PCUN:* precuneus; *PreCG:* precentral gyrus; *SMA:* supplementary motor area; *CAU:* caudate nucleus.

### ROV based brain-behavior correlation and prediction

Brain-behavior relationship was established based on thirteen ROVs showing significant individual variation in the semantic processing network, by using correlation and prediction model respectively. These ROVs can be grouped into functional and structural categories, which is shown in Fig. 3C.

Brain-behavior correlation results indicated that the grey matter volume of the right Angular Gyrus was positively correlated with reading decoding ability (ORRT scores) (r = 0.25, *p* = 0.015) and vocabulary comprehension ability (PVT scores) (r = 0.22, *p* = 0.031) after controlling for brain size variance. These results are shown in Supplementary Fig. 2. The correlations between other ROVs and behavioral scores were not significant after multiple comparison corrections.

**Figure 4:**
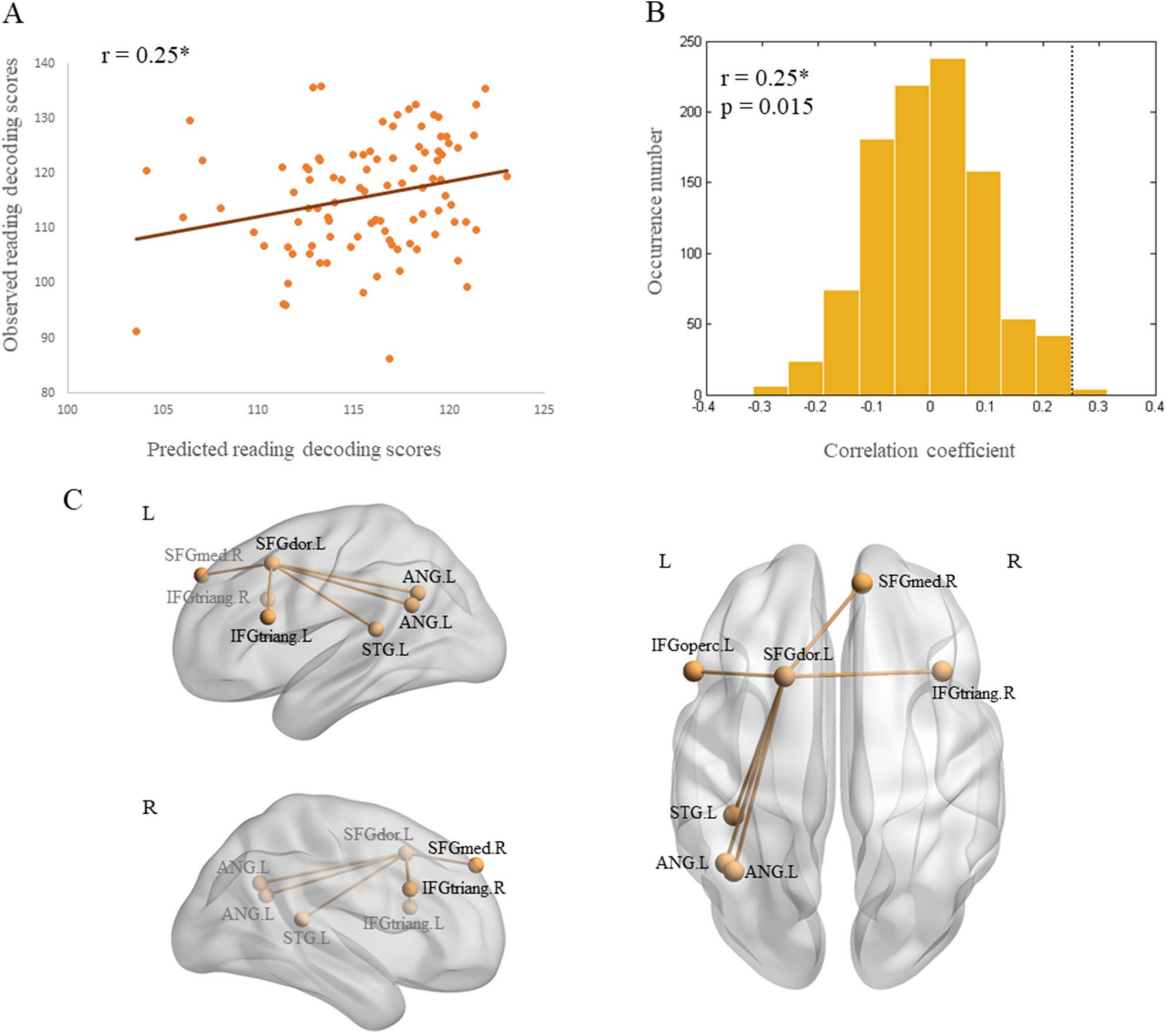
Connectome-based prediction of reading decoding skills and the contributing network distribution. (**A**). Pearson correlation between observed and predicted reading decoding scores. Predicted scores were derived from resting-state FC networks in the training set using a leave-one-out cross validation (LOOCV) method. (**B**). Permutation distribution of the correlation coefficient (r) for the prediction analysis. The correlation coefficient (r = 0.25) between observed and predicted scores of reading decoding are indicated by the dark grey dash line. (**C**). The contributing network in reading decoding prediction. SFGdor: dorsolateral superior frontal gyrus; IFGtriang: inferior frontal gyrus, pars triangularis; IFGoperc: inferior frontal gyrus, pars opercularis; ANG: angular gyrus; STG: superior temporal gyrus; SFGmed: superior medial frontal gyrus. L: left; R: right. BrainNet Viewer (version 1.7) was used to generate brain image (https://www.nitrc.org/projects/bnv/).

A LOOCV approach was implemented to examine whether the task-evoked and resting-state functional networks composed of the thirteen ROVs can predict a novel individual’s language skills (i.e. reading decoding and vocabulary comprehension abilities). Performance of the predictive models was assessed by Pearson correlation between predicted and observed language skills. Results showed that the resting-state positive model was able to predict reading decoding scores in novel individuals (correlation coefficient between observed and predicted scores: r _positive_ = 0.25, *p*_permutation_ = 0.015, see Fig. 4A and B). However, the negative model failed to predict reading decoding abilities because there was no significantly behavior-related edges observed in the correlation matrix between resting-state FC networks and reading decoding scores. Similarly, there was no edges which were significantly correlated with reading decoding abilities observed in the correlation matrix between task-evoked FC networks and reading decoding scores. Hence task-evoked FC networks failed to predict this ability. As for the vocabulary comprehension ability prediction, results showed that the resting-state models failed in prediction (r _positive_ = 0.13, *p*_permutation_ = 0.224; r _negative_ = − 0.06, *p*_permutation_ = 0.548). There were also no significantly behavior-related edges observed in the correlation matrix between task-evoked FC networks and vocabulary comprehension scores.

Based on the resting-state positive model which reliably predicted individuals’ reading decoding abilities, we identified a contributing network in this prediction. This left-lateralized network was composed of seven ROVs, which were distributed across superior, inferior frontal and temporo-parietal cortices, as shown in Fig. 4C. Functional connections were located between the left dorsal superior frontal gyrus and bilateral inferior frontal gyrus, the right medial superior frontal gyrus, the left superior temporal gyrus and angular gyrus. This contributing network was derived from the positive model, hence, the FC strength within it is positively correlated with individuals’ reading decoding skills.

### Individual differences in task-evoked and resting-state functional networks

Individual variance in task-evoked and resting-state functional networks composed of thirteen ROVs were estimated by the across-individual standard deviation (SD) of functional connectivity strength between each pair of ROVs The results are illustrated in Supplementary Fig. 3. The standard deviation of functional connectivity strength in resting state (mean = 0.19) is significantly higher than in task state (mean = 0.05) (T_(77)_ = 26.45, *p* ≤ 0.001).

## Discussion

In this study, we explored how individual differences on neurobiological markers varied throughout a semantic processing network. We confirmed that individual variability in both functional activity and grey matter volume were heterogeneously distributed throughout this network, and increased towards higher levels of the processing hierarchy. In addition, we found that the characteristics of highly variable regions were related with individuals’ language skills. Grey matter volume in the right angular gyrus was positively correlated with reading ability and vocabulary size. Moreover, the intrinsic functional connectivity across the highly variable regions contributed to predict individuals’ reading decoding abilities, and the contributing network was located in left superior, inferior frontal and temporo-parietal cortices. Taken together, these results highlighted the importance for individual differences in both the neural and the cognitive architecture of language.

### Individual differences distribution of local brain activity in the semantic processing network

The first main contribution of this study resides in the finding that the inter-individual variability of task-evoked functional activity was heterogeneously distributed and increased towards higher levels of the semantic processing hierarchy. Specifically, the right medial superior frontal gyrus and left angular gyrus presented the highest individual variance, followed by the left inferior frontal gyrus and right superior temporal gyrus, while the left posterior cingulate gyrus and Heschl’s gyrus showed less variation. Moreover, the right insular and supplementary motor areas failed to show significant variance across individuals although they were significantly activated in the semantic processing network.

In general, the spatial distribution of inter-individual functional variability in the semantic processing network followed the generally same pattern as had been previously reported FC strength during resting state^1,3^, passive movie-watching state^39^, or vocal and non-vocal stimulus listening task^12^, that is, the higher FC variability was observed in multimodal association areas and lower variability in unimodal motor and sensory networks. Although previous efforts have used multiple analysis techniques to define a variety of functional markers, the yielded results are convergent and replicable. Mueller et al. (2013) reported that the spatial distribution of FC variability was correlated with estimated evolutionary cortical expansion, that is, the regions presenting the most prominent individual variability were the regions showing the most rapid expansion during human brain evolution, implying that individual differences in FC might be an outcome of brain size expansion during human brain evolution^1^. Wang and Liu (2014) further illustrated that inter-individual variability was not only closely related to evolutionary expansion, but also to developmental expansion and hemispheric specialization, indicating the highly varied areas also expanded rapidly during human brain evolution, exhibited greater expansion during postnatal development, matured more slowly, and had higher within-hemispheric connectivity than cross-hemispheric connectivity^40^. Furthermore, there are studies reporting that the patterns of individual variability overlapped with the distribution patterns of identification capability, the medial frontal network and the frontoparietal network, both comprised of higher-order association cortices in the frontal, parietal, and temporal lobes, emerged as the most successful in individual subject identification^8^. Taken together, a greatly expanded, slowly maturing, highly specialized, and personalized association cortex can provide more freedom for environmental factors, cognitive and state requirements to act on, and potentially gives rise to individual variability functionally. In contrast, the unimodal sensory and motor processing cortices mature early in life with a low evolution expansion rate, and are more stable to the environmental factors or cognitive task demands, which would present less inter-subject variability.

Besides illustrating the spatial distribution of inter-individual functional variability in a general profile, our data advanced our understanding of a specific profile in semantic processing, by depicting the individual differences distribution of functional activity throughout a semantic network. According to Binder and Desai (2011)’s neuroanatomical model^24^, semantic processing relied on a spatially distributed network of brain regions which were categorized into four components: (1) modality-specific representation zones, located near corresponding sensory, motor, and emotion networks, coding for spatial and temporal configurations of lower-level modal representations; (2) cross-modal high level convergence zones, which are distributed in the inferior parietal lobe and much of the ventral and lateral temporal lobe, bind representations from two or more modalities and store increasingly abstract representations of entity and event knowledge; (3) goal-directed unification zones, which include the dorsomedial and inferior prefrontal cortices, involved in top-down activation and unification/selection of the content stored in convergence zones^41–43^; (4) semantics-memory interface, including the posterior cingulate gyrus and adjacent precuneus, function as an interface between the semantic network and the hippocampal memory system, helping to encode meaningful events into episodic memory. Our data gave further evidence demonstrating that there were regional difference on functional activity in this semantic network, with the highest functional variability occurred in the high-level cross-modal convergence zone and goal-directed unification zone, followed by the semantics-memory interface. However, the modality-specific auditory and motor zones showed the least individual variability. These findings support an argument that the gradient of inter-individual variability may reflect the hierarchy of functional processing; that is, individual differences in functional activity increased as a function of processing hierarchy within the semantic processing network. This argument is supported by a study of Ren et al. (2020), who examined inter-subject variability of functional activity within auditory cortex under a vocal or non-vocal sounds listening task, and demonstrated the individual variability is less in low-level auditory processing regions of Heschl’s gyrus and sulcus but much greater in high-level regions of superior temporal gyrus and planum temporale^44^. Therefore, our results provided further evidence illustrating the individual differences increasing with the processing hierarchy, which is not merely reflected in general, but reflected in the semantic processing domain. Moreover, we elaborated further on the differences in individual variability spatial distribution across the convergence, retrieval zones, and semantics-memory interface, which all belong to the heteromodal association cortex, with the former two zones showing more variance than the semantics-memory interface, locating mainly in posterior cingulate gyrus and precuneus. The posterior cingulate gyrus and precuneus are thought to be one major subdivision of the default mode network (DMN)^45^, has been consistently associated with successful recollection of previous stored items, and presented a significant resting-sate functional connectivity with hippocampal formation, indicating its role in memory and learning^46^. This view about the function of this subdivision of DMN is also consistent with Binder and Desai (2011)’s argument indicating this subdivision helping to encode meaningful events into episodic memory. According to the findings of a study illustrating the relationship between human structural architectures and intrinsic functional networks, structure–function (S–F) correspondence in somatomotor and visual areas was the highest, followed by the DMN and limbic system, while it was the lowest in ventral and dorsal attention network, and frontoparietal network^47^. Luo et al. (2019) interpreted their findings by taking inter-individual variability profile into account, indicating the profile of S–F correspondence was opposite with inter-individual variability: attention and frontoparietal networks with low S–F correspondence presenting the most prominent inter-subject variability^47^. Our results are quite consistent with Luo et al. (2019)’s findings and interpretations by showing the individual variability of DMN was at the median between the frontoparietal and somatomotor network, which might indicating the DMN was not only at the median in S–F correspondences, but also in individual variability of functional activity. In addition, this interesting finding in DMN indicated individual differences in task-evoked brain activity might be mostly driven by task-directed retrieval and storage, other than person-specific and experience-related memories. Future studies might consider incorporating individual variability and S–F correspondence profile and illustrating influence of individual, and task-specific factors to functional brain variability further.

### Individual differences distribution of grey matter volume in the story compression network

Based on the grey matter volume individual variability map, we found the regions with higher individual variability on grey matter volume were mostly distributed in frontoparietal cortices as well, including the left dorsolateral superior frontal gyrus, bilateral inferior frontal gyrus part triangularis, and angular gyrus. The regions with lower individual variability were located in sensorimotor cortices (including pre- and postcentral gyrus), right middle cingulate gyrus, and caudate nucleus. Moreover, right insular cortex and left auditory processing regions (Heschl’s gyrus) failed to show significant variance across individuals although they showed strongest activation in the semantic processing network. These anatomical results agree with our findings of functional activity distribution, showing that high level convergence zones show more individual differences than modality-specific auditory and sensorimotor regions. Studies on whole-brain anatomical variability supported this finding^3,48^. For example, Langs et al. (2016) found that the spatial distribution of association networks, including the frontal, parietal, and temporal regions were highly variable, whereas the unimodal areas, including the motor, sensory, and visual cortices, showed minimal variability in their spatial distribution^48^.

Grey matter volume is a product of two components that are influenced by different factors during development: cortical thickness, which is determined during postnatal development, changes dynamically across the life span as a consequence of development and disease, and hence is more influenced by environment^49,50^; cortical surface area, which is determined during prenatal brain development, and increases during late fetal development as a consequence of cortical folding^49^, and inter-individual variability in cortical folding is primarily established by term birth, changing only modestly thereafter^51^, and hence may be less influenced by environment. Therefore, the non-uniform regional distribution of individual variability in grey matter density may be caused more likely by cortical thickness, which is highly influenced by environment and development, especially in association networks, which show relatively late maturation and hence are more likely to be affected by environmental factors which are characterized by more individual variability. However, further studies are still needed to investigate the roles of cortical thickness and surface area on grey matter volume variability in detail.

Except to verify previous findings about regional distribution of inter-individual anatomical variability in a general profile from grey matter volume perspective, we elaborated grey matter volume variability throughout the semantic network. By taking Binder and Desai (2011)’s interpretation about the semantic network into account, the higher anatomical variability was observed in the top-down goal-directed retrieval zone (left dorsolateral superior frontal gyrus and bilateral triangle part of inferior frontal gyrus) and high-level cross-modal convergence zone (bilateral angular gyrus); the lower variability occurred in motor representation zones and semantics-memory interface; while modality-specific auditory representation zones failed to individual variability. This is mostly consistent with aforementioned functional variability results, that is, both functional and anatomically individual differences increased as a function of processing hierarchy throughout the semantic network with the top-down retrieval and cross-modal storage zone showing the greatest individual differences while the semantics-memory interface, and modality-specific motor or auditory representation zones showing less individual variability. In short, both functional and anatomical individual differences increased towards higher levels in the processing hierarchy throughout the semantic processing network.

### Intrinsic functional connectivity within highly variable regions can predict language skills

Based on the areas which were highly varied in function or structure, we found that grey matter volume of the right angular gyrus was positively correlated with reading decoding ability and vocabulary size. Right angular gyrus acts as a concept integration and semantic knowledge storage zone in the semantic processing network. Therefore, individuals with excellent crystalized intelligence (i.e., vocabulary accumulation and language experience) might have larger grey matter volume in this area.

We further demonstrated that the resting-state functional network composed of the highly variable areas can predict reading decoding abilities across individuals. Specifically, the contributing network was composed of connections between the left dorsal superior frontal gyrus and bilateral inferior frontal gyrus, the right medial superior frontal gyrus, the left superior temporal gyrus and angular gyrus. It is worth noting that all of the nodes in this contributing network were the regions with the largest inter-individual variability. They act as information unification or storage hubs in language processing. These results demonstrated the behavioral significance of the network composed of areas with large inter-individual variation. The importance of variable regions have been reported in previous studies. For example, Omura, Aron, and Canli (2005) introduced a novel approach for selecting Regions of Interest on the basis of their variance characteristics. They demonstrated that their approach yielded the greatest likelihood of capturing the relations between brain and behavior, while minimizing false positive errors^32^. Oudyk, Burunat, Brattico, and Toiviainen (2019) further reported that regions with variation were more related to individual differences in personality, compared to the regions that showed the highest overall activity in whole-brain analyses^21^. However, the way to establish brain-behavior relationship of these studies merely focused on correlation analyses. Our results are the first to demonstrate that the network based on highly varied regions can predict individuals’ reading skills, which ensured prediction model generalizability and individual prediction.

Moreover, this contributing network was derived from the resting-state positive model; that is, the FC strength within this network was positively correlated with individuals’ reading decoding skills. The resting-state functional connectivity among superior temporal gyrus, inferior frontal gyrus, and angular gyrus have been frequently reported to play a role in reading ability^52,53^. The left dorsal superior frontal gyrus was reported to be a goal-directed unification zone, involving in top-down activation and unification/selection of the stored content^41–43^. Its resting-state FC with typical language regions (Wernicke's and Broca's areas) was reported to be able to predict individuals’ reading comprehension accuracy and speed^54^.

Both resting and task-evoked FCs have been used to predict individual cognitive and personality differences in previous studies, but the majority of these works focused on the resting state domain^8,19,55^. However, FCs can also depend on different task demands, task-evoked FCs in individual prediction for cognitive traits, and its comparison with resting-state FCs prediction remain largely unexplored. There was evidence suggesting that the organizations of functional networks were similar at rest and during various tasks, and only moderately modified FC patterns throughout the brain^56^, and hence the prediction effect might be similar across resting and task states. Evidence also indicated that task-evoked brain connectivity promoted the detection of individual differences in brain-behavior relationships^14^. Our results indicated that the task-evoked functional network failed to predict any language skills, and the resting-state functional network could predict individual differences in reading decoding abilities. One potential explanation for this inconsistency is that the functional network in this study is based on highly varied areas, but based on the whole-brain atlas in the previous study. There were no studies comparing these two distinct network construction methods directly, and further studies are needed to give more details to explain this inconsistency. The other potential explanation for this inconsistency is the fact that the less variation across individual task-evoked functional networks. This study indicated that the individual variation of functional connectivity strength in the semantic processing task is significantly lower than in the resting state. This is consistent with previous findings indicating tasks can constrain individual differences in functional connectivity strength^57^. This may be a possibility for the failure of resting-state prediction, but it still needs to be verified in subsequent studies.

### Limitations

Some potential limitations should be mentioned. First, the data set we used in the current study came from the HCP 100 unrelated subjects, which ensures that all participants are not family relatives. This criterion was crucial in our study to exclude the need of family-structure co-variables in our analyses and hence prevented us from using the larger sample datasets. Future studies can be performed on larger study samples to validate these results. In addition, the experimental paradigm in this study is relatively simple, without manipulating any cognitive or psycholinguistic variables during task performance, which might be less refined in interpreting individual variability in language comprehension. This limitation should be addressed in follow-up studies as well. Last, the language-related tests applied in this study is limited to sigle word reading ability and vocabulary size only, further studies should applied more diverse language tests to examine the prediction role of highly varied areas.

## Conclusions

In this study, we investigated the individual variability of functional activity and grey matter volume in a semantic processing network, and demonstrated that the functional network composed of regions with the largest inter-individual variability can predict language skills across individuals. The contribution of this work is twofold. First, for the first time, we reported that that both functional and structural variability profiles were heterogeneous in the semantic processing network, and these variability increased towards higher levels in the processing hierarchy. Second, based on these highly variable retrieval and unification areas, we found the grey matter volume of the right angular gyrus was positively correlated with reading ability and vocabulary size. We further provided novel evidence that the resting-state functional network composed of these highly variable areas can predict reading decoding ability across individuals. The contributing connections were mainly distributed in left superior, inferior frontal and temporo-parietal cortices. This study thus provided relevant insights into functional and anatomical inter-individual underpinnings of the individual variability in semantic processing.

### Supplementary Information

Supplementary Figures.

## Data Availability

The data that support the findings of this study are available on HCP website (https://www.humanconnectome.org/study/hcp-young-adult/document/1200-subjects-data-release), the analyzing code are available on request from the corresponding author.
