# Supplementary material for: Individual differences in the neural architecture in semantic processing
Source: Sci Rep. 2024 Jan 2;14:170. doi: 10.1038/s41598-023-49538-8 (PMC10761854; doi:10.1038/s41598-023-49538-8)
Supplement: Supplementary file 1 — Supplementary Figures. [file 41598_2023_49538_MOESM1_ESM.docx]

**Supplementary materials**

Supplementary Figure 1.


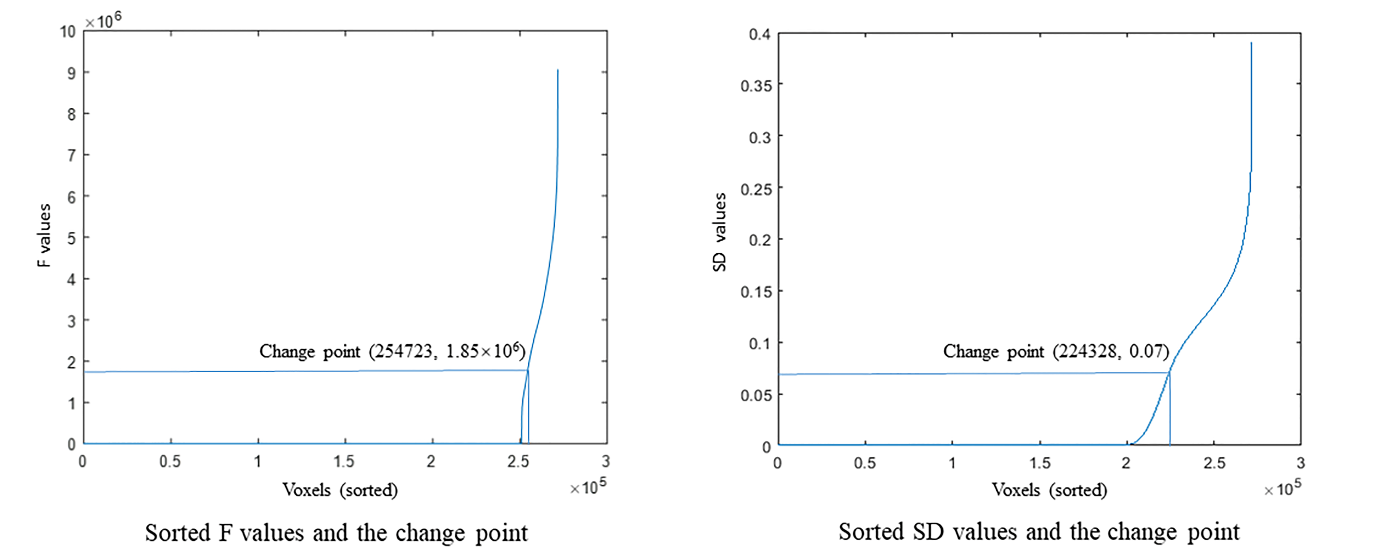


Supplementary Figure 1. Sorted F values in the functional activity variance map (left) and sorted SD values in grey matter density variance map (right), with the corresponding change point in each map, which was used as the cluster-forming threshold for the respective variance map.

Supplementary Figure 2.


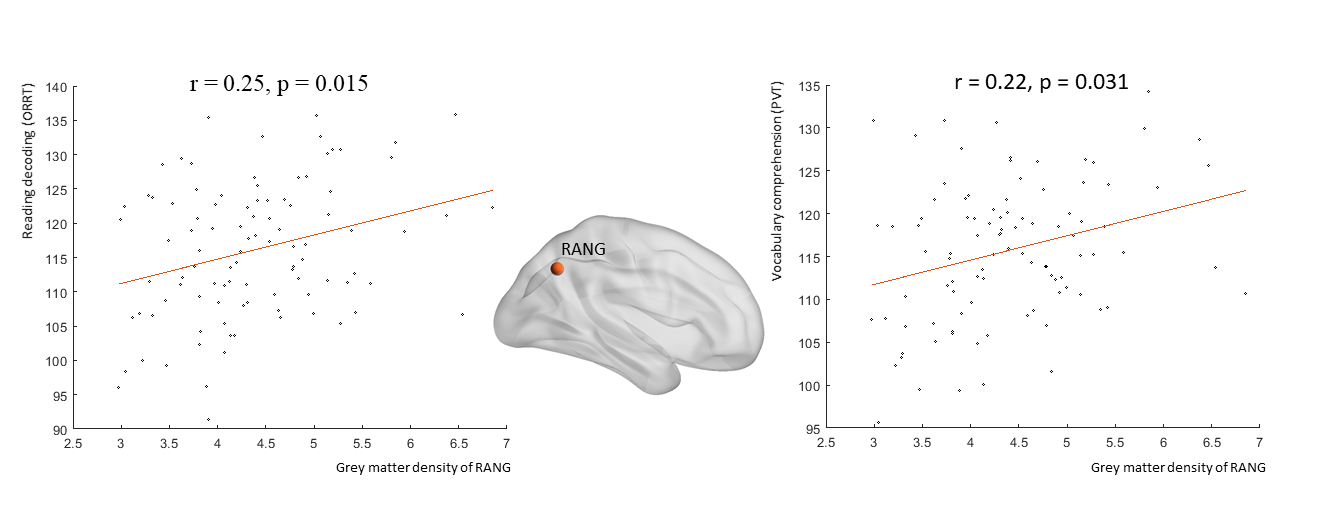


Supplementary Figure 2. Correlations between variability of functional activity, or grey matter volume and language skills. Grey matter volume of RANG was positively correlated with reading decoding ability (ORRT scores) (r = 0.25, p = 0.015) and vocabulary comprehension ability (PVT scores) (r = 0.22, p = 0.031) after controlling for brain size variance.

Supplementary Figure 3.


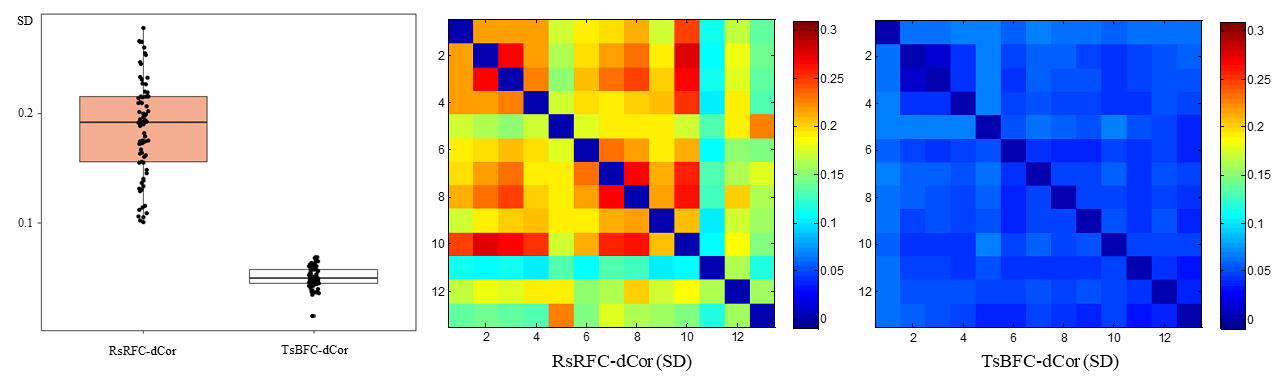


Supplementary Figure 3. Individual differences of the task-evoked and resting-state functional networks composed of thirteen regions of variance (ROVs). TsBFC-dCor (right) refers to the across-individual standard deviations (SDs) in the task-evoked functional network, and RsRFC-dCor (middle) refers to the SDs in the resting-state functional network. The left box plot indicated the across-individual SDs of 78 functional connectivity in resting state (orange) is significantly higher than in task state (white).
